# Draft genome sequences of two thermophilic, spore-forming *Aeribacillus pallidus* strains isolated from dairy products

**DOI:** 10.1128/mra.00896-23

**Published:** 2023-12-13

**Authors:** Genia Lücking, Klement Albrecht, Erwin Märtlbauer, Kristina Schauer

**Affiliations:** 1 Chair of Microbial Ecology, TUM School of Life Sciences, Technical University of Munich, Freising, Germany; 2 Department of Veterinary Sciences, Faculty of Veterinary Medicine, Ludwig-Maximilians University of Munich, Oberschleiβheim, Germany; 3 Department of Microbiology, School of Genetics and Microbiology, Moyne Institute of Preventive Medicine, Trinity College Dublin, Dublin, Ireland; Portland State University, Portland, Oregon, USA

**Keywords:** *Aeribacillus pallidus*, draft genome sequence, spore-forming bacteria, thermophilic lifestyle of bacteria, heat-resistant spores, product spoilage, dairy sector

## Abstract

The presence of thermophilic spore-forming bacteria is challenging in industrial food processing. The presented genome sequences of *Aeribacillus pallidus*, isolated from raw milk and cocoa powder, provide insights into how to prevent damage to minimally processed foods and products with extended shelf life, such as milk products.

## ANNOUNCEMENT

The genus *Aeribacillus* (formerly genus *Geobacillus*) comprises two species, *Aeribacillus pallidus* and *Aeribacillus composti* (1, 2). Many strains of *Aeribacillus* have been isolated from sewage (3), oil-contaminated soil (4), high-temperature oilfields (5), hot springs (6), deep geothermal reservoirs (7), and compost (2). *Aeribacillus pallidus* is a Gram-positive, aerobic, endospore-forming, thermophilic, and alkali-tolerant bacterium with optimal growth at 55°C–60°C and pH 7–9. The ability of thermophilic, endospore-forming bacteria to form resistant spores and biofilms can cause serious challenges in different fields of the food industry. Since spores are able to survive extreme environmental conditions, they can also resist commercial food preservation techniques, e.g., heat treatment, drying, or pressure, resulting in food spoilage of the products (8, 9). *A. pallidus* is ubiquitous in the food industry, and some strains have already been isolated from soy-based and canned (especially corn) food products (8, 10). The dairy industry is also affected in its whole manufacturing chain: *A. pallidus* has been found in dairy cattle (11), raw milk (9, 12), dehydrated ingredients like whey powder (12), and processed milk products like cocoa powder (9). Here, we report the draft genome sequences of two aerobic spore-forming *A. pallidus* strains MHI3390 and MHI3391 isolated from cocoa powder and raw milk associated with industrial dairy processing environments and product spoilage (9).

Both *A. pallidus* strains were isolated as described in reference (9) from autoclaved food matrix (20 min, 100°C) and identified after enrichment in BHI broth (Merk, Germany) for 24 h and cultivation on BHI agar at 55°C by Fourier-transform infrared (FT-IR) spectroscopy (9). Genomic DNA was extracted from overnight cultures in BHI at 55°C using the Monarch^®^ Genomic DNA Purification Kit (New England Biolabs GmbH). Preparation of the DNA libraries was carried out using a DNA Prep Kit (Illumina). The libraries were sequenced by a 150-bp paired-end sequencing on the Illumina NextSeq 1000 machine. Reads were trimmed using Cutadapt v1.16.6 (13) and quality controlled using FastQC v0.72 (https://github.com/s-andrews/FastQC). *De novo* assembly was performed using Unicycler v0.4.6.0 (14), which includes SPAdes v3.12.0 in default mode (15). The NCBI Prokaryotic Genome Annotation Pipeline (PGAP) v5.0 (16) was used for annotation. The codon tree pipeline in PATRIC v3.6.9 (17) was applied for phylogeny confirmation. Default parameters were used for all software unless otherwise specified. All genome informations are presented in Table 1.

**TABLE 1:** Characteristics and accession numbers of genomes from thermophilic, spore-forming *Aeribacillus pallidus* isolated in dairy products

Characteristics	*Aeribacillus pallidus*	
	MHI3390	MHI3391
Origin	Cocoa powder	Raw milk
GenBank accession no.	JAVLRY000000000	JAVLRZ000000000
BioProject no.	PRJNA996925	PRJNA1001175
SRA no.	SRR25462037	SRR25832771
Read count	24,353,451	24,435,450
Genome coverage (×)	275	255
Genome size (bp)	4,146,505	3,888,245
No. of contigs	66	171
N_50_ (bp)	196,125	39,252
G + C content (%)	39.74	39.35
No. of CDSs	3,753	3,640
No. of rRNA operons	9	8
No. of tRNA genes	78	78
CRISPRs	11	9
Plasmid	None	None
Antibiotic resistance	*bla*TEM-116, *vanYE*, *vanTG*, *qacGJ*	*bla*TEM-116, *vanYE*, *vanTG*, *qacGJ*
Bacteriocine clusters	Sactipeptides, *skfA*, lacticin Z, amylocyclin, LAPs^*a*^	Sactipeptides, *skfA*
References (strain name)	(9, 18) (F43)	(9, 18) (F8)

^*a*^ LAPs, linear azole-containing peptides.

Comprehensive Antibiotic Resistance Database (CARD) Resistance Gene Identifier (RGI) analysis predicted the presence of several antibiotic resistance genes (TEM-116 β-lactamase, *vanYE*, *vanTG*) and a multidrug-resistant efflux pump QacGJ (19). A search with the BAGEL4 Web server (20) identified potential bacteriocin gene clusters related to the biosynthesis of sactipeptides, a sporulation killing factor A (*skfA*), a circular bacteriocin amylocyclin, lacticin Z, and linear azole-containing peptides. The genome sequences are expected to provide deeper insights into the thermophilic lifestyle of *A. pallidus* and its ability to form heat-resistant spores, and will contribute to the understanding of the prevalence and impact of *A. pallidus* on the dairy sector.

## Data Availability

The genome sequences of the two thermophilic spore-forming strains have been deposited at DDBJ/ENA/GenBank under accession numbers listed in Table 1.
